# A novel 3D visualization tool for large-scale neural networks

**DOI:** 10.1186/1471-2202-14-S1-P158

**Published:** 2013-07-08

**Authors:** Alexander Jones, Justin Cardoza, Denver J Liu, Laurence C Jayet Bray, Sergiu M Dascalu, Sushil J Louis, Frederick C Harris

**Affiliations:** 1 Department of Computer Science & Engineering, University of Nevada, Reno, Nevada 89557, USA

## 

For neuroscientists, understanding the physiological processes behind memory, learning, and cognition is paramount. By developing computational models of the brain, scientists can simulate how neurons interact with each other to give rise to mental faculties. However, these models often require technical expertise to simulate and are often hard to analyze due to the lack of comprehensive visualization tools. This open source, cross platform software package attempts to abstract the complex setup and analysis of neural simulations by providing a convenient, easy to use graphical interface for managing large-scale models. This tool couples with the Neocortical Simulator (NCS) [1] developed at the Brain Computation Laboratory to allow easy simulation and visualization of neurons and synapses in real time.

Upon starting the application, a user can choose to launch a new simulation or connect to a running simulation given proper login credentials. If choosing to launch a new simulation, the user simply provides a file containing the model to be run and identifies which defined attributes (e.g. firing rates, synaptic strengths, membrane potentials) are to be visualized. Once the model has been specified, the user can manually select what available hardware (CPU & GPU) the simulation should be distributed across or choose to distribute among available hardware using a predefined template. Once the simulation is initiated, the user can render the current network state in 3D, visualizing the complete network hierarchy including synapses, neurons, and networks of neurons. Model defined attributes attached to components of the network hierarchy are represented visually by coloring the components they describe. The user interface provides full customization of all attribute coloration schemes allowing for powerful visualization of the full attribute domain. The application also contains a myriad of additional features (e.g. data logging, video capture, attribute graphing, and camera paths).

**Figure 1:**
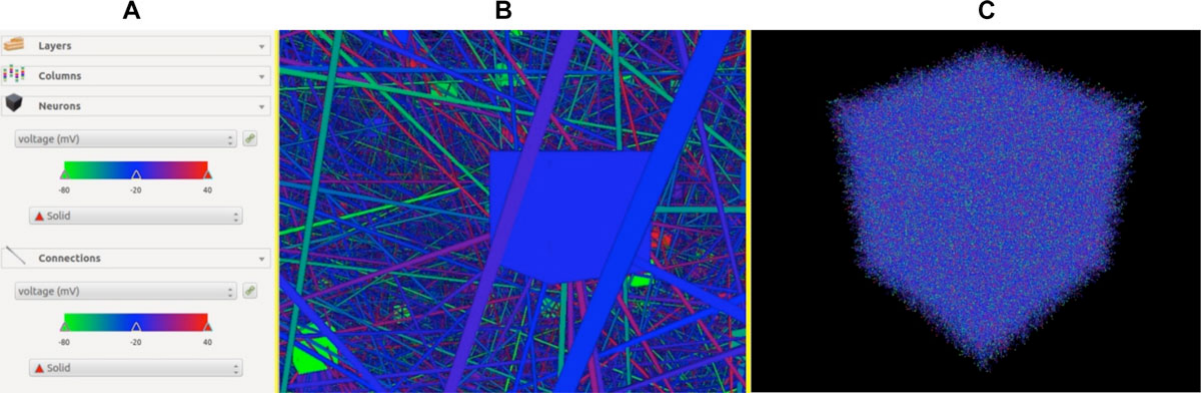
**Visualization Example**. The user interface widget shown in **(A) **dictates an example of the model-defined attributes being rendered for the connections (synapses) and neurons in addition to the current coloration for each attribute. An example of a 50,000 neuron, 250,000 synapse model is shown, close up **(B) **and from a distance **(C)**.

This software is capable of powerfully visualizing neural simulations without requiring expensive computer hardware; it can render large scale simulations (over 100,000 neurons and 250,000 synapses) on some mid-range laptops, as shown in Figure 1. The only current hardware restriction imposed on users is that the host system must contain a modern GPU capable of running at least an OpenGL 3.3 context which is nearly ubiquitous among modern computers. Overall, this novel tool provides an easy way to analyze neural activities and manage large-scale models during real-time simulations.
